# A GABA Interneuron Deficit Model of the Art of Vincent van Gogh

**DOI:** 10.3389/fpsyt.2020.00685

**Published:** 2020-07-13

**Authors:** Federico E. Turkheimer, Erik D. Fagerholm, Miriam Vignando, Jessica Dafflon, Pedro F. Da Costa, Paola Dazzan, Robert Leech

**Affiliations:** Department of Neuroimaging, Institute of Psychiatry, Psychology, and Neuroscience, King’s College London, London, United Kingdom

**Keywords:** interneuron, GABA, psychosis, schizophrenia, Vincent van Gogh, painting, self-portraits, neuroscience of art

## Abstract

Vincent van Gogh was one of the most influential artists of the Western world, having shaped the post-impressionist art movement by shifting its boundaries forward into abstract expressionism. His distinctive style, which was not valued by the art-buying public during his lifetime, is nowadays one of the most sought after. However, despite the great deal of attention from academic and artistic circles, one important question remains open: was van Gogh’s original style a visual manifestation distinct from his troubled mind, or was it in fact a by-product of an impairment that resulted from the psychiatric illness that marred his entire life? In this paper, we use a previously published multi-scale model of brain function to piece together a number of disparate observations about van Gogh’s life and art. In particular, we first quantitatively analyze the brushwork of his large production of self-portraits using the image autocorrelation and demonstrate a strong association between the contrasts in the paintings, the occurrence of psychiatric symptoms, and his simultaneous use of absinthe—a strong liquor known to affect gamma aminobutyric acid (GABA) alpha receptors. Secondly, we propose that van Gogh suffered from a defective function of parvalbumin interneurons, which seems likely given his family history of schizophrenia and his addiction to substances associated with GABA action. This could explain the need for the artist to increasingly amplify the contrasts in his brushwork as his disease progressed, as well as his tendency to merge esthetic and personal experiences into a new form of abstraction.

## Introduction

### Prologue

Over the last two decades, there has been an increasing interest in the relationship between the art and the brain. The focus of this research spans from the investigation of how the brain reacts to art to the attempt to understand the biological underpinning of the artistic experience, with the ultimate aim of deriving more general insights into other cognitive processes. Such an interest has coalesced into two independent areas with some degree of overlap, *neuroesthetics* and *neuroscience of art*. Specifically, neuroestethic experiments have sought to determine the neural underpinnings of esthetic experience, with research including but not being limited to art. In contrast, the neuroscience of art has instead focused on studying the mechanisms underlying human engagement with art, focusing not just on esthetic experience but also on social, semantic, contextual, and evolutionary aspects—for a discussion and review see Pearce et al. (1).

Neuroesthetic studies have explored the neural correlates of human esthetic preference (2); for instance Ticini et al. (3) investigated how congruent priming for brushstrokes positively affects esthetic judgement, Wang et al. (4) explored the neural correlates of mind wandering while attending landscape paintings, while Salimpoor and colleagues attended at the neuroanatomical and neurochemical bases of listening to music (5, 6), to cite a few.

The neuroscience of art has brought forward investigations in the attractiveness of art for humans (7, 8), as well as theories as to what the biological function of art might be, for example by arguing that art is a way to display talent and genetic quality (9, 10).

One of the difficult problems in the neuroscience of art is the understanding of the relationship between the art and the artist. It is straightforward to think that art production is strongly related to higher-order cognitive abilities, such as abstraction and symbolic cognition, but also to lower-level neurochemical processes, such as those at the basis of vision (11). Monet and Cassat had crystalline lens, conditions that alter how the light impact cone cells, and Rembrandt and Titian had ageing-related eye conditions—see (12) for a broader discussion.

In this manuscript we focus on lower-level aspects of the relationship between art and neuroscience by considering a multi-scale model of brain function that we previously published (13) in order to explain the relationship between the illness of Vincent van Gogh and his art. Using the large number of self-portraits produced by the artist during a time frame that spans the development of his illness, we propose that both the micro-features of his brush work (e.g. the color contrast in the paintings) and the increasingly abstract content of these portraits can be explained as the result of a functional deficit of parvalbumin GABA (gamma aminobutyric acid) interneurons. We corroborate this account with contemporary witness reports of a family history of schizophrenia, a disorder linked to GABA interneuron dysfunction (14), and with the demonstration of a quantitative relationship between the color contrast of his self-portraits and the timeline of his use of substances with known pharmacological activity involving GABA receptors.

### The Life of Vincent van Gogh

Vincent van Gogh (1853–1890), one of the most famous painters in the history of Western culture, continues to intrigue clinical and experimental neuroscientists who have looked at possible connections between events of his life, his mental illness, and his unique style.

Born in Zundert, in the south of the Netherlands, the son of a Protestant minister, van Gogh was the first of 6 children. His two brothers, like Vincent, did not reach their 40^th^ birthday while his three sisters lived to an old age—with the youngest, Willemien, spending the latter half of her life in a psychiatric institution suffering from schizophrenia (15). After ending his school education at 15, van Gogh entered the art world as a clerk in an international art dealership and in 1873 he was moved to the London branch. This period became an opportunity for self-development, bringing a fascination of the arts as he frequently visited famous local institutions, such as the British Museum and the National Gallery. However, this period also brought romantic disappointments and loss of interest and disillusionment with the commercial art world leading to his recall to Paris 2 years later and ultimately to his dismissal.

The following years, until 1881, were marked by increasingly erratic behavior and religious fervor. He spent brief periods in England, the Netherlands, and Belgium, becoming increasingly in need of support from his parents and, ultimately, from his youngest brother Theo, who was to become the source of his income for the rest of his life. In fact, even in the most industrious years of his career, van Gogh never sold any painting except for one, the *Red Vineyard at Arles* (16).

While his private life was deteriorating, his mental health also deteriorated with periods of severe depression, a generally gloomy mood that made him renounce any social life, and lead to isolation even from his family, while his interest in art was growing as he dedicated ever more energy toward drawing, sketching, and painting. In 1886, at the age of 33, van Gogh moved to Paris where his younger brother Theo, now manager at the art dealership where he was first employed, introduced him to the colorful work of prominent modern artists like Claude Monet, Henri de Toulouse-Lautrec, Paul Gauguin, and Émile Bernard. It was in Paris that he also started suffering minor panic attacks, temporary lack of consciousness, memory lapses, dystonic postures, and vacant stares (15). Importantly, van Gogh’s illness seemed to be precipitated by his use of absinthe, an alcoholic beverage that was favored by French artists of the time (17). Absinthe is a liquor distilled from an alcoholic steep of herbs, the most significant constituent of which is thujone. In addition to absinthe, it has been reported that van Gogh in later years made use of two other substances, pinene, and camphor that, similar to thujone, are also ketone-terpenes (18). Interestingly, and relevant to the model we propose here, thujone, pinene, and camphor are all modulators of GABA-alpha receptors with the first being a potent antagonist while the others act as mild agonists (19–22).

Two years later, toward the end of the winter of 1888, van Gogh left Paris for the southern city of Arles; there he was joined by Paul Gauguin who was also a keen absinthe drinker (23). The visit lasted a couple of months during which the companionship became increasingly tense and ended in an incident in which van Gogh attacked his friend with a knife (15). The attack was followed by van Gogh’s self-mutilation of his left ear which led to his hospitalization during which he experienced an acute psychotic state with agitation, hallucinations, and delusions (15); this was followed by a year of self-commitment to the asylum in Saint-Rémy (15). He left the mental hospital in May 1890 and spent his last few months in the French locality of Auvers-sur-Oise. However, his illness caught up with him and on the 27^th^ of July 1890, aged only 37 he walked into a wheat-field and shot himself in the chest, still managing to stagger back to his room where he eventually died from the injuries sustained 2 days later.

### Vincent van Gogh: The Illness

Much has been written about the nature of van Gogh’s illness. During the last 10 years of his life he suffered from both visual and auditory hallucinations, delusions, mood swings, committed self-mutilation, and ultimately suicide (15, 23). There have been a number of physiological explanations proposed for his actions, including Ménière’s disease, tertiary syphilis, lead poisoning, intracranial masses, temporal lobe epilepsy, and dementia caused by vascular hypertension; these hypotheses are reviewed in depth, and dismissed, by Cooper and Agius (24) who are inclined to support a diagnosis of schizo-affective disorder. This is a mental disorder that presents with both features of schizophrenia and of bipolar disorder or depression. The proposal that van Gogh might have suffered from a schizo-affective disorder is based on evidence of the combination, on one side, of reactive depression followed by euphoric periods whereas speed of onset and offset of mood changes would have been too quick for a bipolar illness. On the other side, van Gogh was able to achieve total recovery from his psychotic episodes which also seems to rule out schizophrenia (24).

More generally, a point of debate that arises from the literature is whether van Gogh’s unique style can be attributed to a painter who incidentally also happened to suffer from a disease, or whether it was in fact the disease itself that allowed this unique style to emerge (23). In order to answer such a complex question, one approach is to link a potential model for his illness to the art style which he created. To this aim, we turn to a model of brain function we introduced previously (13), which can account for a number of features at different neural scales and, specifically, incorporates the evolving color contrasts in his paintings and the iconic evolution of his art toward abstract expressionism. This model is detailed in (13) and is outlined in the next section.

### A Multi-Scale Model of Brain Function: From the Sensory Cortex to the Default Mode Network

Models provide a simplified construct that allows for complex phenomena to be described and for predictions to be made (25). One key challenge within neuroscience is developing models of brain function that operate across scales from molecular, single cell, cellular ensembles, to macro-observables (elements of tissue large enough to generate signals measurable with EEG or fMRI) and finally to behavior (26). In this context, we previously published a model of brain function (13) that posits the pyramidal interneuron gamma network (PING) as the elementary unit of the cortical system (27). In the PING configuration a pyramidal neuron (PN), when driven by an asynchronous excitatory input, recruits a fast-spiking parvalbumin interneuron (IN), which then synchronizes the output of the PN *via* fast feedback inhibition—generating frequencies in the gamma range. Importantly, the laterally-projecting INs simultaneously inhibit PNs within the same neuronal column or across columns. This lateral inhibition represents the first step in cortical filtering (28). The PING elementary unit can then be extended across spatial scales; every unit functions as an oscillator in cortical space and interacts with adjacent PING units drifting in and out of synchrony, allowing for efficient information processing across spatial scales. The gamma oscillation of PING units acts as the functional glue that allows for neuronal masses of different sizes across cortices and ultimately across the brain itself to communicate (13).

This particular model has interesting properties, one of which is self-similarity across scales which means that the same structural or functional patterns are repeated at increasing space and timescales. The model allows for a parallel to be drawn between the phenomenon of lateral inhibition, observed at the micro-scale between elementary PING units, and the coupled excitation/inhibition activity—observed at the macro-scale between sensory cortices and those areas belonging to the default mode network (DMN). The DMN is a set of brain regions that demonstrate, with high reproducibility, anti-correlated brain activity in respect to the cortical areas that are activated by experiments involving external sensory processing (29). The negatively-correlated activity of the DMN often presents as positively correlated activity when the task involves internalized attention (30).

Interestingly, one can then use the model to predict that any impairment of lateral inhibition at the cellular level will translate to a similar impairment in the synchronization of excitation/inhibition between regions of the DMN and their respective cortices. One may then conjecture that GABA deficits at the level of spiking parvalbumin interneuron would translate across scales to the level of primary sensory cortices, and ultimately into an individual’s inability to resolve contrasts between visual, auditory, and olfactory inputs. Furthermore, DMN dysfunction is associated with a lack of ability to distinguish between internal thoughts and external inputs (13). Interestingly, people with psychosis, a disorder that has been associated with parvalbumin neuron dysfunction (14) on the one hand demonstrate prominent lower-level pathway dysfunction with marked problems in lateral inhibition within the visual, auditory, and olfactory cortices (31–33). Interestingly, John Hyman in his essay on art and neuroscience, has cited as best example of neuroscientific explanations of artistic features, the link between color effects of impressionist painting and lateral inhibition (34).

On the other hand, at the macro-scale, they have a DMNs that exhibits altered temporal frequencies and spatial locations—properties that are in turn associated with difficulties in internal monitoring, as well as with the experience of hallucinations and delusions (35).

### Proposal: GABA Interneuron Deficit as the Neurobiological Underpinning of the Art of Vincent van Gogh

In the case of van Gogh, there is considerable evidence pointing to GABA interneurons as the potential neurobiological underpinning of his mental illness. Schizophrenia was part of his family history, given that his sister Willemien suffered from this disorder for most of her adult life (15). The presence of deficits in gabaergic interneurons in schizophrenia has been supported by evidence from both postmortem and, albeit indirectly and less consistently, *in vivo* studies (36–38). Furthermore, relevant to the history of van Gogh, GABA dysfunction has also been reported by some studies in individuals at risk of schizophrenia, possibly reflecting a vulnerability to this disorder (39, 40). In the case of van Gogh, it is possible that his use of, and possibly addiction to substances such as absinthe, pinene, and camphor, which all interfere with GABA alpha receptors (18), triggered the onset of symptoms on a substrate of vulnerability. Absinthe, a green liquid with an anise smell, is made by distilling a mixture of alcohol, herbs (notably wormwood) and water. It became a national drink in France in the late 19th century. Fashionable among the artistic community, it became cheap enough to be the drink of choice among the poor. While absinthe drinking spread, so did associated health problems. Doctors described symptoms such as addiction, fits, hallucinations (both auditory and visual) and delirium (41). Wilfred Arnold made a detailed study of Van Gogh illness, with careful consideration of all his letters and documents, and purported a diagnose of acute intermittent porphyria (AIP) (23). AIP is a genetic disorder caused by low levels of the enzyme porphobilinogen deaminase involved in the synthesis of hemoglobin. Attacks of AIP bring about symptoms such as vomiting, diarrhea, pains in the back and abdomen, physical weakness and mental problems, and are usually precipitated by alcohol or drug consumption or stress. Symptoms of AIP have been attributed to its metabolite, delta-aminolevulinic acid (ALA) that selectively competes for the binding of GABA to synaptic GABA receptors in central nervous system membranes (42). In his 1988 paper, Arnold noted the dates of van Gogh acute crises and linked them to his absinthe consumption (23).

The involvement of interneurons in the illness of van Gogh is not a novel concept; Taghipour et al., have recently proposed that an early brain injury (which would also be supported by the significant craniofacial asymmetry evident in his portraits) of a possible hypoxic nature may have damaged his hippocampal basket cell interneurons and caused temporal lobe epilepsy (43). However, this theory is in contrast with what asserted by Cooper and Agius (24), e.g., that epilepsy was a viable diagnosis at the time and that treatments were available, but none were ever administered to him.

Here, we use the multi-scale model of brain function outlined above to support the involvement of GABA parvalbumin interneurons in the cortex and specifically we wish to:

1. demonstrate, at the micro-scale, through a temporal and quantitative analysis of van Gogh self-portraits, that variations in his brushwork were associated with changes in his ability to perceive visual contrasts and with those periods when exacerbation of his illness was associated with his consumption of thujone analogs;
2. use the model, at the macro-scale, to provide a more general explanation as to how an interneuron-deficit disorder may have opened an unexplored avenue that led to abstract art. We use recent results in the neuroscience of art that have demonstrated the central role of the DMN in intense esthetic experiences (44–46). We then construct a qualitative argument to explain how a dysfunctional relationship between the DMN and visual cortices may actually fuse the artistic (internal) and the esthetic (external) aspects of perception that result in an experience consistent with the creation of abstract art.

## Methods

### The Self-Portraits of Vincent van Gogh

van Gogh painted over 30 self-portraits in just 3 years, between 1886 and 1889, a series that fully reflects his ongoing pursuit of complementary color contrasts and bolder composition. His collection of self-portraits places him among the most productive self-portraitists of all time. This production also spans the period comprising the start of his illness in Paris in 1886, its worsening until the Gauguin incident in Arles, the period of recovery in hospital, up until the commitment to the asylum in Saint-Rémy prior to his death in 1890.

**Figure 1:**
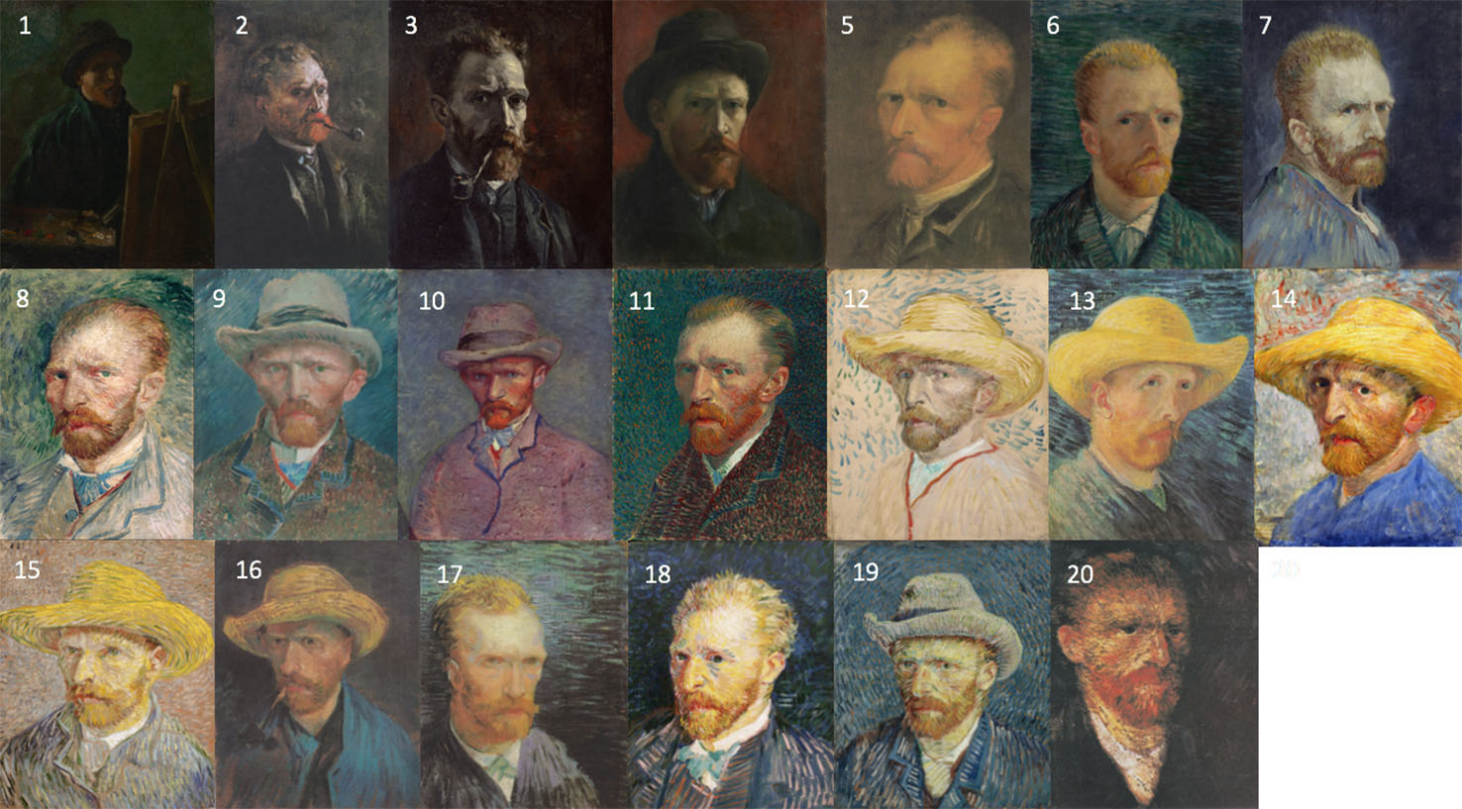
A chronological list of self-portraits painted by van Gogh during his stay in Paris between 1886 and 1888 (Credits: Van Gogh Museum, Amsterdam - Vincent van Gogh Foundation).

**Figure 2:**
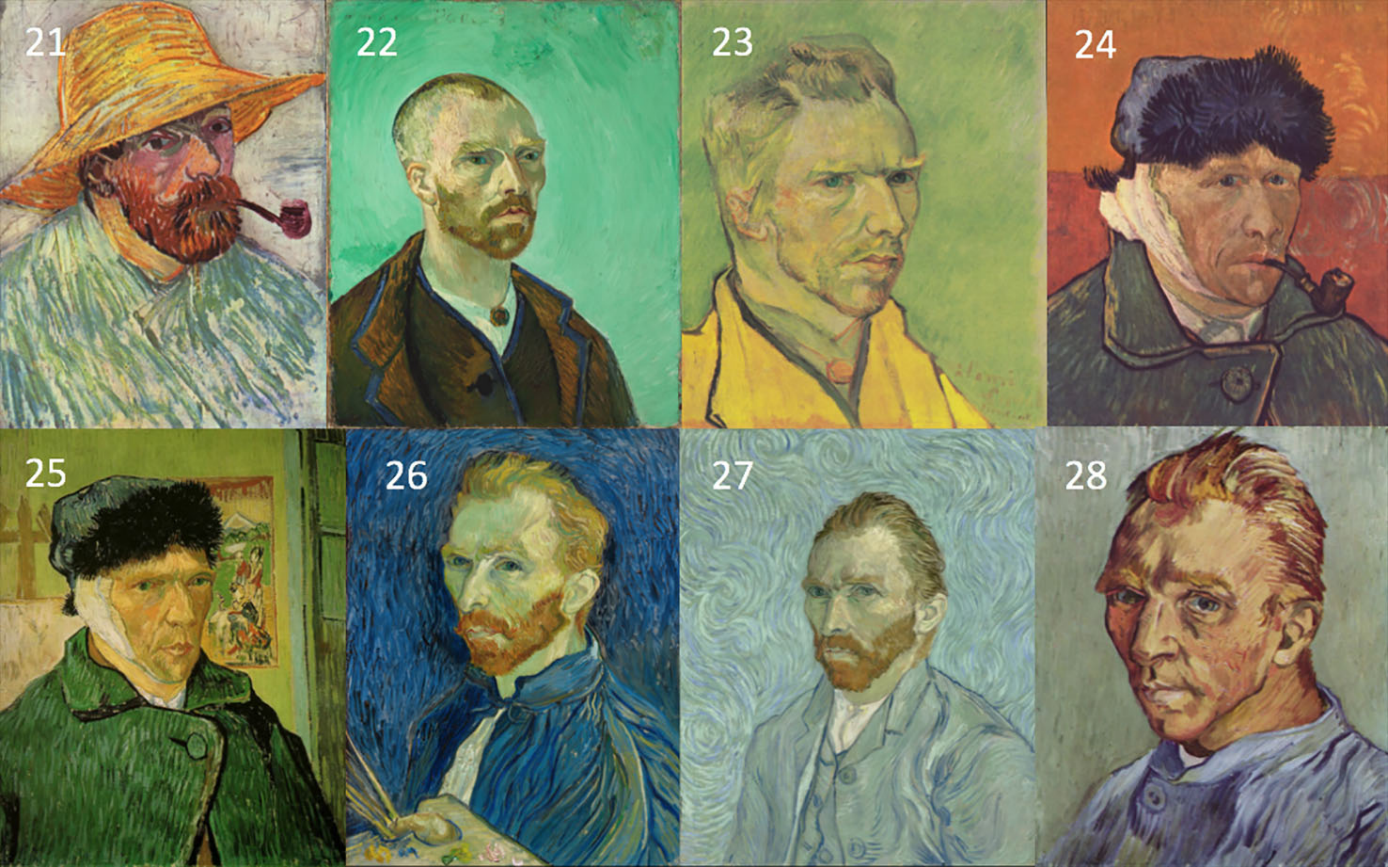
A chronological list of self-portraits painted by Vincent van Gogh during his stay in Arles and at the asylum of St-Remy between 1888 and 1889. The sequence is numbered continuing from the portraits in **Figure 1** (Credits: Van Gogh Museum, Amsterdam - Vincent van Gogh Foundation).

Self-portraits are useful for quantitative analysis since the subject matter remains relatively fixed over time, and the predominant source of variability is, instead, the artistic style. The self-portraits considered here were all sourced together with details on the dates of production, from the digital collection available at the Van Gogh Museum in Amsterdam (https://www.vangoghmuseum.nl/en/search/collection). Of the available self-portraits, 28 were selected for analysis as they were available as digital plates at high resolution, 20 of which were painted in Paris between 1886 and 1888 (**Figure 1**). The remaining 8 were painted between 1888 and 1889 during his stay in Arles and also in the asylum of Saint-Rémy (**Figure 2**).

In order to test the fitness of our proposed model to the data, given the effect of GABA dysfunction on contrast perception, we aimed to obtain a measure of contrast from these images with an expectation of contrast increases in times of excessive absinthe intake. For each digital image, the luminance of a pixel was obtained from its red, green, blue (RBG) components as 0.299R + 0.587G + 0.114B (47).

In order to quantify the contrast of the luminance in the portraits, we considered the digital image as a 2-dimensional random process which is fully characterized by its mean and its autocorrelation function. The autocorrelation function describes the relationship (correlation) between two samples of a random process at a certain lag. The autocorrelation function evaluated at its origin is the average normalized power in the random process, e.g. the average contrast across the image (48). The computationally efficient approach to the calculation of the autocorrelation function of a N-dimensional process exploits the Wiener–Khinchin theorem, that states that the autocorrelation function of a wide-sense-stationary random process has a spectral decomposition given by the power spectrum of that process (49). The latter can be quickly calculated for a 2D-process using the 2-D Fourier Fast Transform (FFT) (49); hence the matrix of luminance values was transformed using the 2D-FFT and the value at the origin produced the required average image contrast. All calculations were performed using Matlab (v. R2018b, The Mathworks Inc., Natick, MA, USA).

Average image contrast was then tested between two conditions, low absinthe consumption and high absinthe consumption (portraits 9-10-11-12-21-22-23-27-28) where conditions were defined according to absinthe consumption reported in Table 1 of (23). Difference in consumption between the two conditions was tested using a Mann-Whitney U test using SPSS v.25 (IBM Corp).

## Results

**Figure 3:**
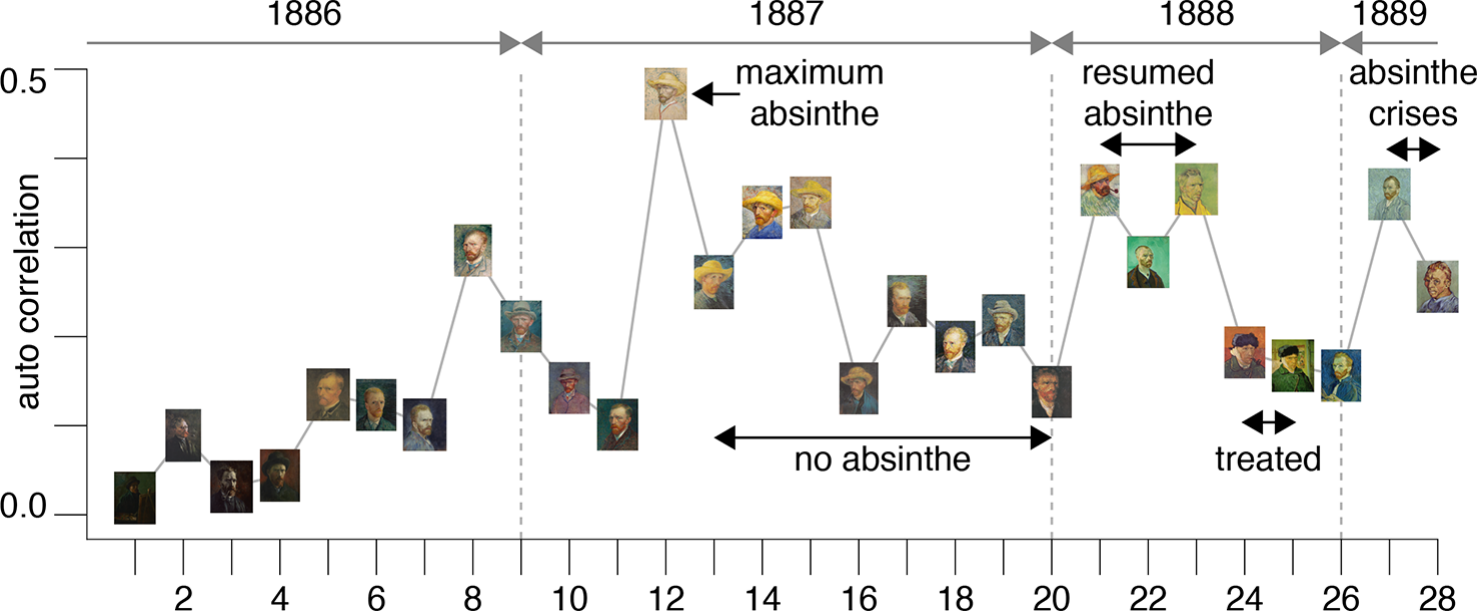
The plot shows the average image contrast of the self-portraits; the sequence follows the numbered order of the previous figures.

The results are plotted in **Figure 3** and illustrate an interesting timeline that one could also appreciate through the qualitative analysis of the self-portraits in **Figures 1** and **2**. The average image contrast of the paintings starts at low levels, close to 0, for the early production in Paris in 1886, as this coincides with a style that resembles the late 19^th^ century standard (16). The contrast then steadily increases, reaching the maximum level ~0.5 in the first months of 1887 (portrait no. 12). We can relate the variability of the image contrast with events in the life of van Gogh. The famous painting *Café Table with Absinthe*, that became the symbol for the consumption of this alcoholic drink by the Parisian artistic elite, was in fact painted by van Gogh in February-March 1887 (16), in a period that coincided with the first serious symptoms of his illness, including episodes of sudden terror, lapses of consciousness, irascibility (15). Soon after, his consumption of the liquor stopped, only to resume when he arrived in Arles in 1888 (15). It is again unlikely to be a coincidence that the image contrast in the self-portraits drops in the second half of 1887 (portraits no. 13-20), only to recover in 1888 (portraits 21-23), which is when his drinking, as well as his use of other substances, restarted and the illness took a stronger hold, ultimately causing him to have delusions and hallucinations that prompted the attack on Gauguin.

This theory is further corroborated by the fact that the self-portraits numbered 24-25, that have much lower image contrast, show the artist with a bandage on his ear lobe, a self-inflicted injury after the attack on Gauguin; these portraits were painted while he was receiving treatment. The last bouts of his disease (late 1889 early 1890) before his death were all triggered by absinthe and are accurately documented by (23); there are only two self-portraits (numbered 27-28) for this period of time. It is clear from the plot in **Figure 3** that the contrast for these works increased again to the very high levels characterizing the previous severe periods of his illness. Considering the entire timeline, the difference in image average contrast between the low- and high-absinthe consumption states as defined above was statistically significant (p=0.016).

All of the above is consistent with the hypothesis that the changing style of van Gogh’s brushwork was closely associated with substance abuse. In other words, the consumption of thujone analogs that likely impaired his parvalbumin interneuron function possibly caused the artist to increase the contrasts in his work in order to compensate for problems in lateral inhibition in his visual cortices.

## Discussion

From his start in Paris in 1886, van Gogh’s emerging style saw him display an ever-increasing emotional expression on subjects through his use of patterns and brushwork. He deliberately used colors to capture mood, rather than using them realistically, unlike any other artist at the time. This is well captured by the words of the artist himself: *”Instead of trying to reproduce exactly what I see before me, I make more arbitrary use of color to express myself more forcefully”* (Letter 663 to his brother Theo, Arles August 1888, van Gogh Museum, Amsterdam).

The intensity of artistic experiences has been the subject of recent work in the neurosciences. Vessel, Starr, and Rubin (45) were the first to use fMRI to show that esthetic experience engages sensory regions, resulting in functional activity in occipito-temporal regions and in increased activity of the striatum, linearly with esthetic ratings of the paintwork. However, their study also reports a stepwise increase in activity in another set of regions that activated specifically in response to the piece of art that individuals rated as ranking at the top of their appreciation scale. This network mostly contains regions of the DMN (45). This work has since been replicated (45, 50) and supports the idea that art and in particular abstraction (i.e., separating a depiction from any literal, representational point of reference and merging it with the personal) requires the combined activity of sensory cortices and the DMN—two sets of brain networks typically viewed as functioning in opposition to one another (50).

As hypothesized, a dysfunction in GABA interneurons is likely to cause problems in the alternating activation and deactivation of the DMN and its adjoining cortices. In the case of van Gogh, we propose that this phenomenon might have facilitated the biological underpinning of the fusion between the esthetic and personal experiences.

In this manuscript we used a multi-scale model of brain function to test the hypothesis that van Gogh’s illness may have exerted important influences upon his artistic creations. This was achieved by first analyzing the micro-features of his art in a quantitative analysis by using the average image contrast of his paintings, and secondly by inspecting the macro-features of his work in a qualitative fashion. However, it would be naïve to presume that the unique style of van Gogh can be attributed largely to his illness. The features discussed are not informative with respect to the qualitative value of his creations, nor do they suggest that impaired function of parvalbumin interneurons could be the cause of greater artistic achievement. Interestingly though, similar quantitative approaches have been used to try to understand the phenomenon of artistic appreciation. In the only other application of a quantitative model to van Gogh’s paintings, Aragon and colleagues (51) demonstrated that luminance fluctuations in a number (but not all) of the most famous of van Gogh’s paintings, for example *Starry Night*, result in a statistical distribution that lies close to that of turbulent flow. Turbulence is characterized partly by the existence of energy cascades between the largest and smallest scales. They did this by using the entire shape (and not just the value at the origin as reported here) of the autocorrelation function; this function denotes the relationship between the luminance at increasing distances between the voxels of the paintings and in the case of *Starry Night* this function was a logarithmic decay. This is interesting because the same logarithmic decay has been observed in the autocorrelation function of structural brain images and characterizes both the time and space correlation at rest in human functional activity when measured with EEG, MEG or fMRI (52). The similarity between the autocorrelation function of the art piece and the functional response to it by the observer has been indicated as the potential mechanism underlying the appreciation of painters (53) with specific examples such as Jackson Pollock (54) (but see also (55) for a quantitative comparison between Pollock and van Gogh), as well as of composers, such as Bach and Mozart (56–58).

## Limitations

The study presented here has some limitations. Firstly, this is an historical investigation and the data portion of this work demonstrates an association between the image contrast of van Gogh’s self-portraits and his absinthe consumption; the latter was not directly measured but derived indirectly from reports and, in particular, the detailed study of Arnold [Table 1 of (23)]. Hence, this is meant as another part of the tapestry of data concerning the relationship between the art of van Gogh and his mental illness but ultimately, given the lack of post-mortem reports, this relationship will never be fully elucidated; hopefully though, it will inform future studies in this area.

Secondly, one needs to be aware that Van Gogh’s paintings could have undergone changes in luminescence over time, for example with regard to chemicals used or the interaction with the material used (59). This may have contributed to some of the variation in the data presented in **Figure 3**. Consider for example the portrait no.15 in **Figure 3** [titled “Self-portrait with straw hat” (60)]. For this work, Van Gogh used cardboard as a less expensive alternative to canvas and then applied a layer of oil for priming with dashes of purple. The purple pigment in the paint has largely faded over time lessening the contrast with the yellow straw; however, this portrait still holds the largest average contrast of the series in **Figure 3**.

Finally, we chose as quantitative metric the average image contrast, a simple tool derived directly from our model of interneuron dysfunction with consequent disturbance in lateral inhibition; more sophisticated analytical approaches have been proposed that can dissect different aspects of van Gogh’s style (see for example [61)] although not necessarily related to the biology of his disorder.

## Conclusion

In summary, we suggest that the timeline of the image contrast across the self-portraits of van Gogh highlights interesting clues as to the potential biological bases of his disorder, and we hope that this work will be of value with respect to the study of the relationship between psychiatric disorders and artistic creation.

## Data Availability Statement

The datasets generated for this study are available on request to the corresponding author.

## Author Contributions

All authors contributed to the article and approved the submitted version. FT and PD performed the data analysis. All agreed to be accountable for all aspects of the work, its accuracy and integrity.

## Funding

FT wishes to acknowledge support from a project grant from the UK Medical Research Council (Ref: MR/K022733/1). EF and RL were funded by the MRC (Ref: MR/R005370/1). The authors would also like to acknowledge support from the Data to Early Diagnosis and Precision Medicine Industrial Strategy Challenge Fund, UK Research and Innovation (UKRI), the National Institute for Health Research (NIHR), the Biomedical Research Centre at South London, the Maudsley NHS Foundation Trust, and King’s College London.

## Conflict of Interest

The authors declare that the research was conducted in the absence of any commercial or financial relationships that could be construed as a potential conflict of interest.
